# Impact of Non-Dialysis-Requiring Acute Kidney Injury on Survival Outcomes in Non-critically Ill Hospitalized Medical Patients in a Resource-Limited Setting: A Retrospective Cohort Study

**DOI:** 10.7759/cureus.69358

**Published:** 2024-09-13

**Authors:** Nahom Dessalegn Mekonnen, Tigist Workneh Leulseged, Yared Adane Minas, Zekarias Tadele Alemneh, Yonas Hailu Gebeyehu, Thomas Tadesse Meshesha, Mebratu Abera Gurara, Betelhem Tiruneh Gebremedhin, Nathnael Tesfa Lisanu, Bezawit Woldaregay Wagaye, Mowlid Bedel Ahmed

**Affiliations:** 1 Internal Medicine, St. Paul’s Hospital Millennium Medical College, Addis Ababa, ETH; 2 Public Health, Medical Research Lounge, Addis Ababa, ETH; 3 Internal Medicine, St. Paul's Hospital Millennium Medical College, Addis Ababa, ETH; 4 General Medicine, Médecins Sans Frontières Belgium (Doctors Without Borders), Addis Ababa, ETH; 5 General Medicine, Kalba Hospital, Emirates Health Services, Sharjah, ARE; 6 Medicine, Addis Ababa University, Addis Ababa, ETH; 7 Radiology, St. Paul's Hospital Millennium Medical College, Addis Ababa, ETH; 8 General Medicine, ALERT Comprehensive Specialized Hospital, Addis Ababa, ETH; 9 General Medicine, Jigjiga University Sheik Hassen Yabare Comprehensive Specialized Hospital, Jigjiga, ETH

**Keywords:** ethiopia, medical patients, non-critical patients, non-dialysis requiring acute kidney injury, retrospective cohort, survival analysis

## Abstract

Introduction

The severe consequences of acute kidney injury (AKI) have been well-documented in high-risk patient populations. However, the effects of milder forms in non-critically ill patients remain understudied, particularly in resource-limited settings. While the risk of mortality associated with these cases is considered low, it can still lead to various complications including prolonged hospitalization, which may influence long-term renal and patient survival. Hence, the objective of this study was to study the impact of non-dialysis-requiring AKI (NDR-AKI) on survival outcomes of non-critically ill medical patients admitted to St. Paul’s Hospital Millennium Medical College in Ethiopia during the period from July 2019 to January 2022.

Methods

A retrospective cohort study was conducted among 300 non-critically ill medical patients, 93 with NDR-AKI and 207 without AKI. Descriptive statistics, including frequency distributions and median survival times, were employed to summarize the data. Kaplan-Meier curves and the log-rank test were utilized to compare survival experiences of groups. A Cox proportional hazards survival model was fitted to estimate the impact of NDR-AKI on time to recovery. Adjusted hazard ratio (AHR) with 95% confidence interval (CI) was used to report findings.

Results

Two hundred four (68.0%) were discharged after improvement and the median recovery time was 16 days (95%CI: 13.5-18.5 days). Having NDR-AKI was associated with a 43% lower rate of achieving recovery (AHR=0.57, 95%CI=0.38, 0.84, p-value=0.004). Females were found to have a 1.41 times higher rate of recovery (AHR=1.41, 95%CI=1.03,1.94, p-value=0.033). Additionally, having tuberculosis (AHR=0.41, 95%CI=0.23,0.72, p-value=0.002) and being on anticoagulant (AHR=0.67, 95%CI=0.47,0.95, p-value=0.027) were associated with a 59% and 33% lower rate of recovery, respectively.

Conclusion

NDR-AKI significantly delays recovery compared to patients without AKI suggesting that even milder forms of AKI in non-critically ill patients can negatively impact patient outcomes. Early identification, prompt management, and addressing underlying causes are key to improving recovery and reducing long-term morbidity and mortality. Strict screening and monitoring of high-risk groups such as men, patients with tuberculosis, and those on anticoagulants is also crucial.

## Introduction

Acute kidney injury (AKI) is a serious medical condition that affects more than 10 million people worldwide each year [1]. Although it can occur in the community, it has been found to be a common complication among hospitalized patients and is in turn linked to a higher likelihood of both immediate and long-term adverse health outcomes, including morbidity and mortality [2-5].

The substantial impact of AKI and its associated complications is well-established in the literature, with a particular emphasis on high-risk patient populations, including individuals with severe AKI requiring renal replacement therapy (RRT) and those admitted to critical care settings, due to the serious consequences associated with these cases. These patients are shown to have a high mortality rate of up to 23.9%, with a 10-fold increased risk as compared to those with no AKI [6-10]. This risk is reportedly even higher in resource-limited settings, with an increased risk of death reaching up to 36.9% [11-14]. However, a few studies conducted in non-critically ill patients also demonstrated that AKI is a prevalent complication occurring in up to 25% of cases [15-17]. This is in turn associated with a range of adverse clinical outcomes, including a four-fold increase in risk of in-hospital mortality, particularly among those with severe AKI [16,17].

Despite the growing body of research on AKI, there remains a dearth of studies specifically focused on milder forms of AKI, particularly in non-critically ill patients. Moreover, evidence regarding outcome indicators other than mortality, such as hospitalization duration, is even more scarce. While the risk of morbidity and mortality associated with mild AKI in non-critically ill patients is considered low, it can still lead to various complications beyond increased mortality, including prolonged hospitalization [15,17]. Delayed recovery, in turn, may influence long-term renal and patient survival due to an increased risk of other organ damage from immune dysfunction, impaired drug clearance, fluid and electrolyte imbalances, and increased susceptibility to hospital-acquired infections. Additionally, patients with mild AKI may face heightened morbidity not only from the disease itself but also from the lack of strict monitoring in the non-critical care setting, especially in an underdeveloped healthcare infrastructure, exacerbating the risk of worse outcomes [18,19].

Understanding the effect of milder forms of AKI in non-critically ill patients is crucial as it steers the development of preventive and therapeutic interventions tailored to these patient populations. This is particularly vital as these patients are known to exhibit a more favorable response to intervention, potentially mitigating further harm to the patient and alleviating the strain on an already overburdened healthcare system, especially in resource-limited settings. Therefore, the aim of this study was to assess the impact of non-dialysis-requiring AKI (NDR-AKI) on survival outcomes in non-critically ill hospitalized medical patients admitted to St. Paul’s Hospital Millennium Medical College (SPHMMC) in Ethiopia between July 2019 and January 2022.

## Materials and methods

Study setting and design

A hospital-based retrospective cohort study was undertaken from September 25, 2022 to January 20, 2023 among non-critically ill medical patients who were admitted to SPHMMC between July 2019 and January 2022. The cohort was classified based on the patients’ diagnosis of AKI on admission (no AKI and NDR-AKI). SPHMMC is one of the largest tertiary referral hospitals in Ethiopia and has been the only national renal transplant facility since 2015. The renal unit functions with eight nephrologists, four fellows and over 70 nurses. The medical ward has 47 beds available for admissions and serves around 850 patients every year.

Population and sample size

**Figure 1:**
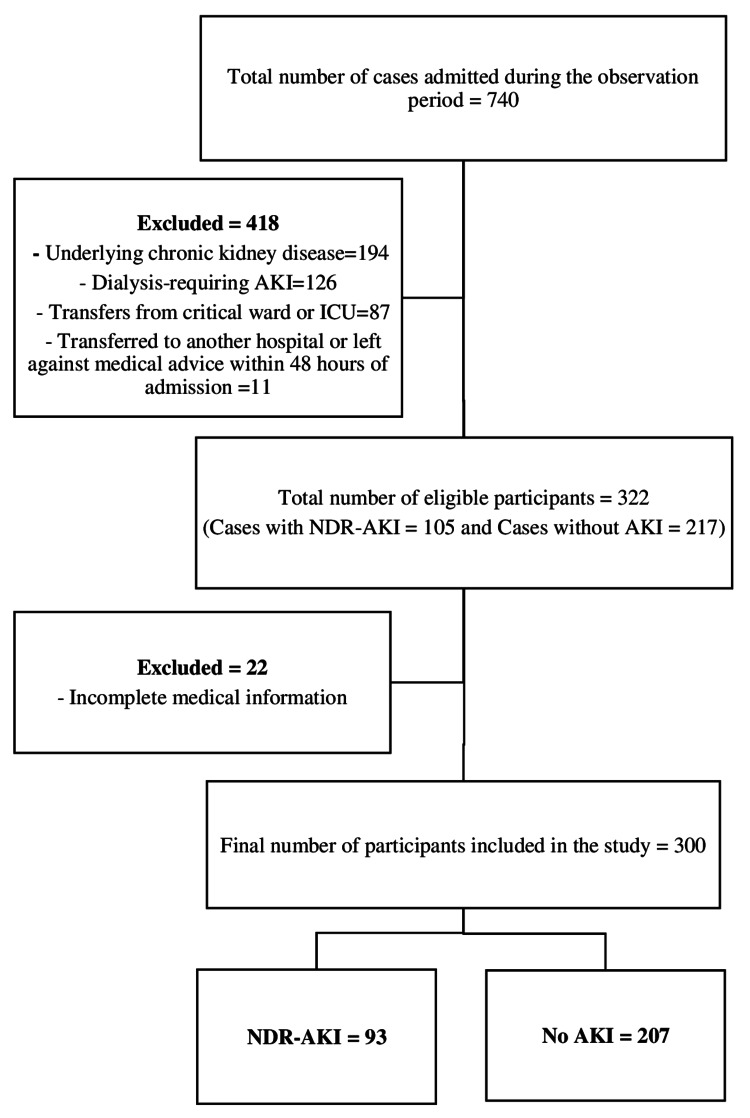
Flow chart illustrating the enrollment of study participants, St. Paul’s Hospital Millennium Medical College (SPHMMC), Ethiopia, from July 2019 to January 2022. AKI: Acute Kidney injury, ICU: Intensive Care Unit, NDR-AKI: Non-Dialysis-Requiring Acute Kidney Injury

The study incorporated all eligible non-critically ill medical patients hospitalized between July 2019 and January 2022. During this period a total of 740 cases were admitted to the medical ward. This is a relatively lower rate compared to the hospital's average annual admissions. The decrease was due to the coronavirus disease 2019 (COVID-19) pandemic, as patients who tested positive were isolated in a separate ward dedicated to their care. Non-critically ill medical patients were defined as those who, while requiring hospitalization, did not exhibit immediate or life-threatening conditions that necessitated admission to the critical care unit during their hospitalization. Additionally, patients were considered eligible if they had no underlying chronic kidney condition at the time of hospitalization, were not transferred to or from the critical care unit during their stay, were not transferred to another hospital or left against medical advice within 48 hours of admission, and had a comprehensive medical record documenting key exposures and outcome variables. In addition, for those with diagnosis of AKI, cases that required dialysis at diagnosis or during follow-up were further excluded. Accordingly, a total of 300 eligible cases were identified and enrolled in the study, 93 patients with NDR-AKI and 207 patients without AKI (Figure 1).

Operational definition

NDR-AKI

AKI that doesn’t require dialysis is characterized by an increase in serum creatinine of at least 0.3mg/dl within 48 hours or a rise in serum creatinine to at least 1.5 times the baseline level, which is known or presumed to have occurred within the preceding seven days but doesn’t result in the need for kidney replacement therapy. Based on the increase in serum creatine, it can be classified into three stages [20].

Stage 1: A rise in serum creatinine to between 1.5 and 1.9 times the baseline level, or an increase in serum creatinine by at least 0.3 mg/dL, or a decrease in urine output to less than 0.5 mL/kg/hour for six to 12 hours.

Stage 2: A rise in serum creatinine to between 2.0 and 2.9 times the baseline level, or a decrease in urine output to less than 0.5 mL/kg/hour for at least 12 hours.

Stage 3: A rise in serum creatinine to at least 3.0 times the baseline level, or an increase in serum creatinine to at least 4.0 mg/dL, or a decrease in urine output to less than 0.3 mL/kg/hour for at least 24 hours, or anuria for at least 12 hours, without the need for initiating kidney replacement therapy.

Event

Recovery from the medical condition is declared when a patient achieves improvement and is discharged. This was diagnosed when the primary medical condition for which hospitalization was required has fully recovered or stabilized to a level that does not require admission care, and any accompanying AKI (for patients with AKI) has resolved or is stabilized to a level that does not require admission care.

Censoring

Patients who died, were transferred to another facility, left against medical advice, or completed their follow-up period without recovering from their medical condition were considered as censored.

Time to Event or Censoring

This was the time from hospital admission to discharge with improvement or censoring (measured in days).

Data collection procedures and quality assurance

Patient medical charts were reviewed using a pre-tested data collection tool to extract information on exposures and outcomes. A team of three trained General Practitioners, supervised by a senior internal medicine resident, collected the data. Data quality was maintained through checks for inconsistencies using frequency and cross-tabulation analyses, and cross-referencing with medical charts. In addition, data cleaning and management were performed to address numeric errors and missing values.

Statistical analysis

Descriptive statistics, including proportions presented in frequency tables and median time to recovery, were utilized to summarize the data. Kaplan-Meier (KM) survival curves were employed to compare the survival experiences of different groups. The log-rank test was used to evaluate the statistical significance of any of the differences between the groups based on the KM results.

To estimate the impact of NDR-AKI on time to recovery, a Cox proportional hazards (PH) survival model was utilized. Variable selection for inclusion in the final model was made using a univariate analysis at a 25% significance level where crude hazard ratio (CHR) with 95% confidence intervals (CI) were used to measure the degree of association. The selected variables from the univariate analysis were subsequently incorporated into the final multivariable Cox PH survival model at a 5% significance level. Adjusted hazard ratios (AHR), 95% CI for AHR, and p-values were used to interpret the results from the final model. The proportional hazards assumption underlying the Cox PH model was verified using the log-minus-log (LML) function, with the plot indicating a satisfactory fit to the assumption through parallel lines between groups, suggesting proportionality. All data management and analysis were performed using STATA software version 17.0, 2021 (College Station, TX, USA).

Ethics approval and consent to participate

The study was conducted after obtaining ethical clearance from SPHMMC-IRB. SPHMMC-IRB also waived the need for informed consent since the study used secondary data (Ref. No. PM23/385). To protect participant privacy, the research report only used medical record numbers, omitting any other personally identifiable information. Data access was limited to the research team, safeguarding confidentiality throughout the study.

## Results

Baseline clinical characteristics and survival experience

The study included 300 eligible non-critically ill medical patients, with 207 cases with no AKI and 93 cases diagnosed with NDR-AKI. From the 93 NDR-AKI cases, 44 (47.3%) had stage 1 AKI, 16 (17.2%) had stage 2 AKI, and the rest (33, 35.5%) had stage 3 AKI. The comparability of the cohorts was analyzed using chi-square and Fischer’s exact test and it was found that patients with NDR-AKI had a significantly higher proportion of patients with hypertension (54.8% vs. 36.2%, p=0.003), and sepsis (18.3% vs. 9.2%, p=0.025). Conversely, a significantly lower proportion of malignancy (3.2% vs 11.1%, p=0.025) and no cases of central nervous system (CNS) infections (8.7% vs. 0, p=0.003) were documented in this group. Additionally, a significantly higher proportion of these patients were taking diuretics (63.4% vs. 25.6%, p-value<0.0001), angiotensin-converting enzyme inhibitors/angiotensin receptor blockers (ACEIs/ARBs) (29.0% vs. 17.4%, p-value < 0.0001), and proton pump inhibitors (PPIs) (76.3% vs. 53.6%, p-value = 0.026). Furthermore, a significantly lower proportion of these patients had normal hemoglobin (33.3% vs. 47.3%, p-value=0.026), potassium (63.4% vs. 80.7%, p-value < 0.0001) and chloride (48.4% vs 66.2%) levels on admission (Appendix).

The majority of the participants were younger than 40 years old (39.3%) and male (55.0%). Upon admission, the most prevalent underlying chronic medical conditions were hypertension in 126 (42.0%) and type 2 diabetes mellitus (T2DM) in 52 (17.3%). The top five most common admission diagnoses were pneumonia in 109 (36.3%), heart failure in 74 (24.7%), deep vein thrombosis/pulmonary embolism (DVT/PE) in 46 (15.3%), sepsis in 36 (12.0%), and stroke in 31 (10.3%).

The survival experience of the patients was compared based on their baseline clinical characteristics, including NDR-AKI, for the presence of a statistically significant difference in the median time to recovery. Accordingly, patients with NDR-AKI showed a prolonged time to recovery compared to those without AKI (20 days vs. 14 days, p-value <0.0001). Additionally, sex and having tuberculosis (TB) demonstrated significant associations where male patients (18 days vs. 14 days, p-value=0.012) and those with TB (30 days vs. 15 days, p-value=0.004) displayed a significantly delayed time to recovery (Table 1).

**Table 1:** Baseline clinical characteristics, censoring status, and comparison of survival experience among non-critically ill hospitalized medical patients at St. Paul’s Hospital Millennium Medical College (SPHMMC), Ethiopia, from July 2019 to January 2022 (n=300) HIV: Human Immunodeficiency Virus, TB: Tuberculosis, DVT: Deep Vein Thrombosis, PE: Pulmonary Embolism, NDR-AKI: Non-Dialysis-Requiring Acute Kidney Injury, CNS: central nervous system. *=Statistically significant at p-value ≤ 0.05.

Variable		Disease outcome		Total N (%)	Median time to recovery (in days)	P-value
		Recovery N (%)	Censored N (%)			
Age Category	<40	79 (66.9)	39 (33.1)	118 (39.3)	15.0	0.977
	40-59	69 (74.2)	24 (25.8)	93 (31.0)	16.0	
	>=60	56 (62.9)	33 (37.1)	89 (29.7)	17.0	
Sex	Male	106 (64.2)	59 (35.8)	165 (55.0)	18.0	0.012*
	Female	98 (72.6)	37 (27.4)	135 (45.0)	14.0	
Hypertension	No	121 (69.5)	53 (30.5)	174 (58.0)	16.0	0.603
	Yes	83 (56.9)	43 (43.1)	126 (42.0)	15.0	
Diabetes	No	169 (68.1)	79 (31.9)	248 (82.7)	15.0	0.828
	Yes	35 (67.3)	17 (32.7)	52 (17.3)	18.0	
HIV	No	193 (67.7)	92 (32.3)	285 (95.0)	15.0	0.349
	Yes	11 (73.3)	4 (26.7)	15 (5.0)	23.0	
TB	No	189 (68.6)	86 (31.4)	274 (91.3)	15.0	0.004*
	Yes	16 (61.5)	10 (38.5)	26 (8.7)	30.0	
Cardiovascular disease	No	192 (68.3)	89 (31.7)	281 (93.7)	16.0	0.741
	Yes	12 (63.2)	7 (36.8)	19 (6.3)	14.0	
Chronic lung disease	No	183 (67.1)	90 (32.9)	273 (91.0)	15.0	0.825
	Yes	21 (77.8)	6 (22.2)	27 (9.0)	16.0	
Malignancy	No	188 (68.6)	86 (31.4)	274 (91.3)	16.0	0.183
	Yes	16 (61.5)	10 (38.5)	26 (8.7)	13.0	
Sepsis	No	184 (69.7)	184 (30.3)	264 (88.0)	15.0	0.748
	Yes	20 (55.6)	20 (44.4)	36 (12.0)	16.0	
Heart failure	No	155 (68.6)	71 (31.4)	226 (75.3)	16.0	0.572
	Yes	49 (66.2)	25 (33.8)	74 (24.7)	14.0	
Pneumonia	No	126 (66.0)	65 (34.0)	191 (63.7)	15.0	0.269
	Yes	78 (71.6)	31 (28.4)	109 (36.3)	18.0	
Hepatitis	No	192 (69.3)	85 (30.7)	277 (92.3)	15.0	0.069
	Yes	12 (52.2)	11 (47.8)	23 (7.7)	26.0	
CNS infection	No	191 (67.7)	91 (32.3)	282 (94.0)	16.0	0.877
	Yes	13 (72.2)	5 (27.8)	18 (6.0)	16.0	
Stroke	No	182 (67.7)	87 (32.3)	269 (89.7)	16.0	0.787
	Yes	22 (71.0)	9 (29.0)	31 (10.3)	15.0	
DVT/PE	No	168 (66.1)	86 (33.9)	254 (84.7)	16.0	0.629
	Yes	36 (78.3)	10 (21.7)	46 (15.3)	13.0	
NDR-AKI	No	156 (75.4)	51 (24.6)	207 (69.0)	14.0	<0.0001*
	Yes	48 (51.6)	45 (48.4)	93 (31.0)	20.0	

Baseline treatment history, laboratory parameters and survival experience

The most frequently prescribed medications were cephalosporins in 234 (78.0%), PPIs in 182 (60.7%), anti-coagulants in 167 (55.7%), vancomycin in 118 (39.3%), diuretics in 112 (37.3%), and steroids in 90 (30.0%). At baseline, more than half of the participants showed one or more abnormal laboratory values for white blood cell count (WBC) (35.3%), hemoglobin (Hg) (57.0%), sodium (Na) (36.7%), potassium (K) (24.7%), and chloride (Cl) levels (39.3%).

A comparison of the median time to recovery was made based on the patients’ medication exposure and laboratory parameters. Accordingly, a significantly delayed time to recovery was observed among patients taking cephalosporin (17 days vs. 13 days, p-value=0.042), vancomycin (19 days vs. 14 days, p-value=0.021), anti-TB (31 days vs. 15, p-value<0.0001), ACEI/ARBs (20 days vs. 15 days, p-value=0.017), and anticoagulants (17 days vs. 14 days, p-value=0.004) (Table 2).

**Table 2:** Baseline treatment history and laboratory parameters, censoring status, and comparison of survival experience among non-critically ill hospitalized medical patients at St. Paul’s Hospital Millennium Medical College (SPHMMC), Ethiopia, from July 2019 to January 2022 (n=300) TB: Tuberculosis, ACEI: Angiotensin-Converting Enzyme Inhibitor, ARBs: Angiotensin Receptor Blockers, PPIs: Proton Pump Inhibitors, WBC: White Blood Cell, Hg: Hemoglobin, Na: Sodium, K: Potassium, Cl: Chloride. *=Statistically significant at p-value ≤ 0.05.

Variable		Disease outcome		Total N (%)	Median time to recovery (in days)	P-value
		Recovery N (%)	Censored N (%)			
Cephalosporin	No	53 (80.3)	13 (19.7)	66 (22.0)	13.0	0.042*
	Yes	151 (64.5)	83 (35.5)	234 (78.0)	17.0	
Penicillin	No	190 (67.4)	92 (32.6)	282 (94.0)	15.0	0.624
	Yes	14 (77.8)	4 (22.2)	18 (6.0)	16.0	
Macrolides	No	170 (66.9)	84 (33.1)	254 (84.7)	15.0	0.199
	Yes	34 (73.9)	12 (26.1)	46 (15.3)	16.0	
Quinolones	No	197 (68.2)	92 (31.8)	289 (96.3)	16.0	0.154
	Yes	7 (63.6)	4 (36.4)	11 (3.7)	14.0	
Vancomycin	No	132 (72.5)	50 (27.5)	182 (60.7)	14.0	0.021*
	Yes	72 (61.0)	46 (39.0)	118 (39.3)	19.0	
Anti-TB	No	187 (68.5)	86 (31.5)	273 (91.0)	15.0	<0.0001*
	Yes	17 (63.0)	10 (37.0)	27 (9.0)	31.0	
Diuretics	No	141 (75.0)	47 (25.0)	188 (62.7)	15.0	0.103
	Yes	63 (56.2)	49 (43.8)	112 (37.3)	16.0	
ACEI/ARBs	No	169 (71.3)	68 (28.7)	237 (79.0)	15.0	0.017*
	Yes	35 (55.6)	28 (44.4)	63 (21.0)	20.0	
Antiplatelet	No	189 (67.3)	92 (32.7)	281 (93.7)	16.0	0.962
	Yes	15 (78.9)	4 (21.1)	19 (6.3)	15.0	
PPIs	No	82 (69.5)	36 (30.5)	118 (39.3)	14.0	0.181
	Yes	122 (67.0)	60 (33.0)	182 (60.7)	17.0	
Anticoagulants	No	86 (64.7)	47 (35.3)	133 (44.3)	14.0	0.004*
	Yes	118 (70.7)	49 (29.3)	167 (55.7)	17.0	
Steroids	No	141 (67.1)	69 (32.9)	210 (70.0)	16.0	0.669
	Yes	63 (70.0)	27 (30.0)	90 (30.0)	16.0	
WBC	Normal	130 (67.0)	64 (33.0)	194 (64.7)	18.0	0.137
	Deranged	74 (69.8)	32 (30.2)	106 (35.3)	13.0	
Hg	Normal	91 (70.5)	38 (29.5)	129 (43.0)	15.0	0.052
	Deranged	113 (66.1)	58 (33.9)	171 (57.0)	16.0	
Na	Normal	136 (71.6)	54 (28.4)	190 (63.3)	15.0	0.865
	Deranged	68 (61.8)	42 (38.2)	110 (36.7)	17.0	
K	Normal	162 (71.7)	64 (28.3)	226 (75.3)	15.0	0.372
	Deranged	42 (56.8)	32 (43.2)	74 (24.7)	16.0	
Cl	Normal	126 (69.2)	56 (30.8)	182 (60.7)	15.0	0.904
	Deranged	78 (66.1)	40 (33.9)	118 (39.3)	17.0	

**Figure 2:**
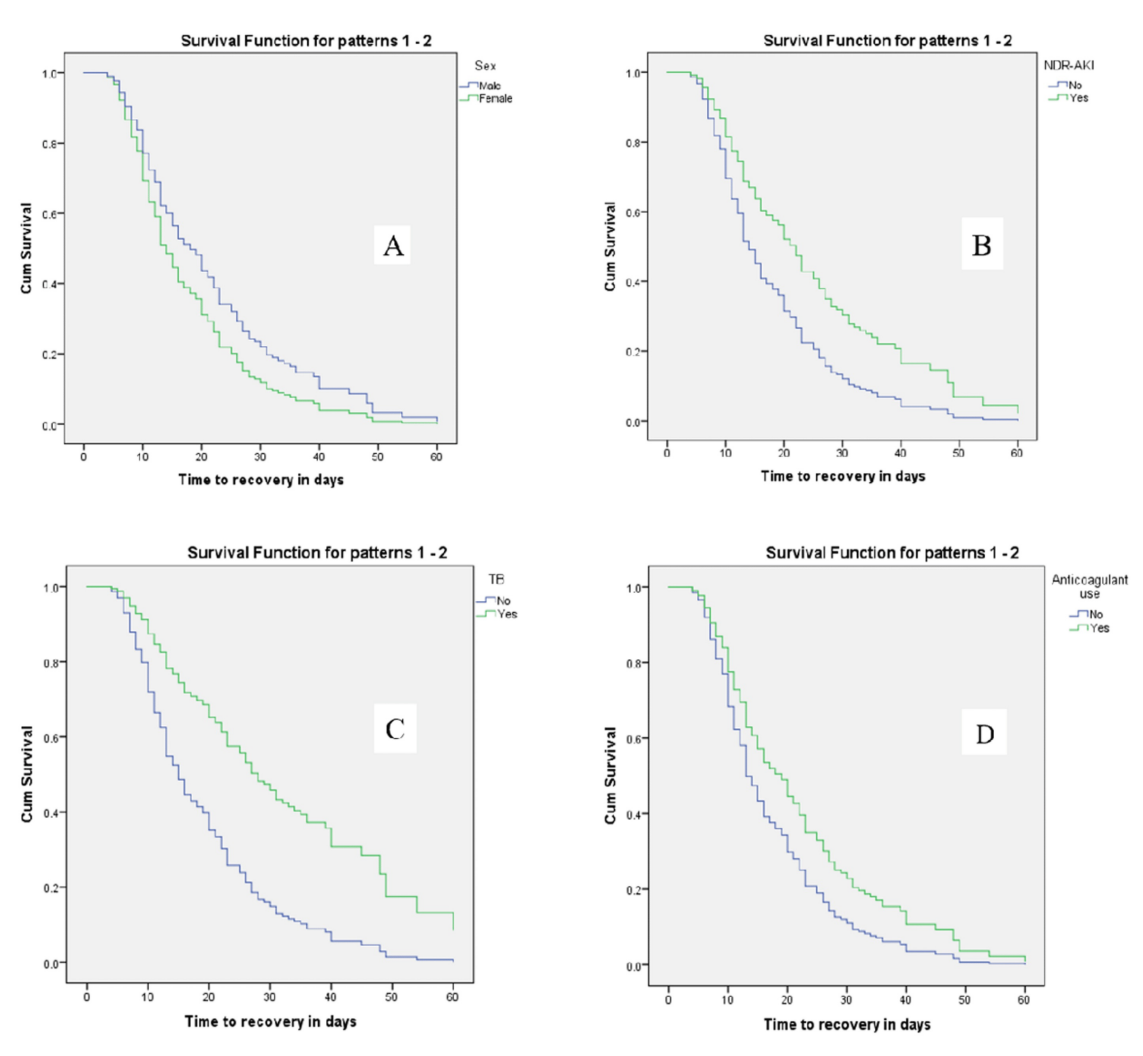
Kaplan-Meier survival curve of time to recovery from medical illness stratified by sex (A), NDR-AKI (B), TB (C), and anticoagulant use (D) among non-critically ill hospitalized medical patients at St. Paul’s Hospital Millennium Medical College (SPHMMC), Ethiopia, from July 2019 to January 2022 NDR-AKI: Non-Dialysis-Requiring Acute Kidney Injury, TB: Tuberculosis

The KM survival function graph also showed that being male, having AKI and tuberculosis, and taking anticoagulants were associated with prolonged recovery time throughout the observation period(Figure 2).

Overall median time to recovery

Among the 300 patients, 204 (68.0%) were discharged with improved medical condition, while the remaining 96 (32%) patients were censored. Of the censored cases, 63 (21.0%) were discharged home without full medical recovery, and 33 (11.0%) died. The total median time to recovery was 16 days (95% CI, 13.5-18.5 days).

Predictors of time to recovery

To determine the impact of NDR-AKI on time to recovery, a multivariable Cox PH model was run controlling for sociodemographic, clinical, treatment, and laboratory-related confounders identified as significant in univariate analysis and judged to be clinically relevant. Accordingly, after adjusting for these covariates, patients with NDR-AKI experienced a 43% decreased rate of achieving recovery compared to those who did not develop AKI (AHR=0.57, 95% CI= 0.38, 0.84, p-value=0.004).

Furthermore, sex, TB and being on anticoagulant were also found to have a significant impact on time to recovery. Females were found to have a 1.41 times higher rate of recovery compared to males (AHR=1.41, 95% CI= 1.03,1.94, p-value=0.033). Additionally, having TB was associated with a 59% lower rate of recovery (AHR=0.41, 95% CI= 0.23,0.72, p-value=0.002) and being on anticoagulant was associated with a 33% lower rate of recovery (AHR=0.67, 95% CI=0.47,0.95, p-value=0.027) (Table 3).

**Table 3:** Predictors of time to recovery among non-critically ill hospitalized medical patients at St. Paul’s Hospital Millennium Medical College (SPHMMC), Ethiopia, from July 2019 to January 2022 (n=300) T2DM: Type 2 Diabetes Mellitus, TB: Tuberculosis, DVT: Deep Vein Thrombosis, PE: Pulmonary Embolism, NDR-AKI: Non-Dialysis-Requiring Acute Kidney Injury, Hg: Hemoglobin, Na: Sodium, K: Potassium, CHR: Crude Hazard Ratio, AHR: Adjusted Hazard Ratio. *=statistically significant at p-value ≤ 0.05.

Variable	CHR (95% CI)	AHR (95% CI)	P-value	
				Age Category (in years) (R: <40)				
40-59	0.97 (0.70-1.35)	1.13 (0.78-1.64)	0.523	
>=60	0.96 (0.67-1.36)	1.09 (0.69-1.73)	0.712	
Sex (Female vs. Male)	1.41 (1.07-1.86)	1.41 (1.03-1.94)	0.033*	
Hypertension (Yes vs. No)	1.08 (0.81-1.42)	1.16 (0.80-1.70)	0.434	
T2DM (Yes vs. No)	0.96 (0.67-1.39)	0.99 (0.65-1.52)	0.970	
Malignancy (Yes vs. No)	1.40 (0.83-2.34)	1.27 (0.73-2.20)	0.397	
TB (Yes vs. No)	0.49 (0.29-0.82)	0.41 (0.23-0.72)	0.002*	
Sepsis (Yes vs. No)	1.08 (0.68-1.72)	0.91 (0.55-1.52)	0.722	
Heart failure (Yes vs. No)	0.91 (0.66-1.26)	0.76 (0.49-1.17)	0.211	
Pneumonia (Yes vs. No)	0.86 (0.64-1.14)	0.76 (0.52-1.12)	0.165	
Hepatitis (Yes vs. No)	0.59 (0.33-1.07)	0.66 (0.35-1.25)	0.201	
Stroke (Yes vs. No)	0.94 (0.60-1.47)	0.77 (0.43-1.37)	0.371	
DVT/PE (Yes vs. No)	1.09 (0.76-1.57)	1.07 (0.69-1.66)	0.774	
NDR-AKI (Yes vs. No)	0.57 (0.41-0.79)	0.57 (0.38-0.84)	0.004*	
Cephalosporin (Yes vs. No)	0.73 (0.53-0.99)	0.92 (0.61-1.40)	0.692	
Macrolides (Yes vs. No)	1.27 (0.87-1.83)	1.14 (0.74-1.76)	0.542	
Vancomycin (Yes vs. No)	0.72 (0.54-0.96)	1.00 (0.69-1.44)	0.985	
Diuretics (Yes vs. No)	0.79 (0.58-1.06)	0.91 (0.61-1.35)	0.627	
ACEI/ARBs (Yes vs. No)	0.65 (0.45-0.94)	0.90 (0.57-1.42)	0.646	
PPIs (Yes vs. No)	0.83 (0.63-1.10)	0.93 (0.68-1.29)	0.683	
Anticoagulants (Yes vs. No)	0.67 (0.50-0.89)	0.67 (0.47-0.95)	0.027*	
Steroids (Yes vs. No)	0.94 (0.69-1.27)	0.99 (0.71-1.39)	0.970	
Hg (Deranged vs. Normal)	0.77 (0.58-1.01)	0.91 (0.67-1.24)	0.545	
Na (Deranged vs. Normal)	0.98 (0.73-1.31)	1.02 (0.75-1.40)	0.894	
K (Deranged vs. Normal)	0.86 (0.61-1.21)	1.06 (0.71-1.58)	0.771	

## Discussion

This study assessed the impact of NDR-AKI on survival outcomes of non-critically ill hospitalized medical patients admitted to a large tertiary referral hospital in Ethiopia between July 2019 and January 2022. The study included 300 eligible patients (93 patients with NDR-AKI and 207 patients without AKI). The comparison of the cohorts demonstrated that the group with NDR-AKI had a moderately higher underlying risk as compared to those with no AKI. Patients with NDR-AKI had a higher proportion of cases with hypertension, sepsis, taking diuretics, ACEIs/ARBs, PPIs, and deranged laboratory values for Hg, K, and Cl. On the other hand, the proportion of patients with malignancy and CNS infection was lower in these groups. Some of these differences between the groups can be attributed to the pathophysiologic changes associated with AKI. Fluid and electrolyte imbalance as a result of AKI may cause hypertension, which could explain why more patients in the AKI group had higher blood pressure [21]. Deranged potassium levels may also be caused as part of the electrolyte imbalance caused by AKI [22]. Studies have also shown anemia, a derangement in hemoglobin, to result as a consequence of AKI due to progressively falling levels of erythropoietin [23]. 

From the 300 patients, 204 (68.0%) were discharged with improved medical condition, while the remaining 96 (32%) patients were censored and the overall median recovery time was 16 days (95% CI, 13.5-18.5 days). The effect of NDR-AKI on time to recovery showed that patients with NDR-AKI took significantly longer time to recover (median of 20 days) than those without AKI (median of 14 days), demonstrating a less favorable survival experience on the KM plot. This finding was further supported by the Cox PH model, which revealed that NDR-AKI patients had a 43% lower rate of achieving recovery compared to those without AKI. Although it is believed that milder forms of AKI are not considered to lead to worse outcomes and that few studies also showed that milder forms of AKI are associated with no increased risk of in-hospital mortality, the possibility of increased morbidity, including prolonged hospitalization, and long-term mortality is demonstrated in some reports [17-19]. This could be because of the fact that even in cases with less severe underlying causes, AKI can worsen other existing medical conditions and complicate the course of treatment due to the kidney's multitude of functions and that its function is related with most organs in the body. Hence any degree of damage to the kidneys causes toxic metabolite accumulation, fluid and electrolyte imbalance resulting in damage of organs, such as the heart, brain and lungs. AKI also increases the risk of infection and bleeding, not to mention the fact that it worsens underlying conditions that cause it in the first place such as shock, liver or heart failure leading to a longer hospital stay [24,25]. Although all potential confounders are controlled in the model, the disparity in the underlying risk between the groups could also result in prolonged hospital stay in the group with NDR-AKI and hence partly account for the significant difference. 

Furthermore, sex, TB, and anticoagulant use significantly affected recovery time. Females displayed a 1.41 times faster recovery rate than males. This could be attributed to a complex interplay of biological differences and disparities in behavioral and social factors. Additionally, males often carry a heavier burden of chronic medical conditions like hypertension and diabetes, and the associated cerebrovascular accidents which necessitate extended hospital stays [26,27].

Having TB was associated with a 59% lower rate of recovery. This is likely due, in part, to the severity of the disease, which often requires longer and more complex treatment regimens. Additionally, these patients may face further complications due to concomitant medical conditions, such as HIV and DM, or adverse drug reactions from the treatment, necessitating prolonged hospitalization [27,28].

Finally, being on anti-coagulant was associated with a 33% lower rate of recovery. Patients who require anticoagulants often have serious underlying medical conditions like clotting disorders, heart disease, or stroke risk. These conditions themselves can necessitate prolonged hospitalization for management and treatment. In addition to that, patients on anticoagulants require regular blood tests to monitor their bleeding time and clotting factors. Furthermore, anticoagulants can also increase the risk of bleeding which can lead to further complications resulting in further prolonged hospitalization, especially if medication adjustments are needed [29,30].

Despite its retrospective nature, the study provides valuable insights to our understanding of the topic as little is known about it. However, it is important to note that additional potential confounders such as behavioral factors, stage and control level of medical conditions, and additional investigation results were not controlled for in the study because of inability to access such information from the medical charts of the patients due to the retrospective design of the study. Furthermore, the relatively small sample size of the study and the single-center design could limit the generalizability of the findings to similar contexts only. Hence, the findings should be interpreted cautiously in light of these limitations.

## Conclusions

The study revealed a significantly longer time to recovery and a lower recovery rate among patients with NDR-AKI compared to those without AKI implying that even milder forms of AKI in non-critically ill patients can negatively impact patient outcomes. Therefore, early identification and prompt management of NDR-AKI, along with addressing underlying causes, are pivotal to improve patient recovery and potentially reduce long-term morbidity and mortality. Furthermore, strict screening and monitoring of high-risk groups, including men, TB patients, and those on anticoagulants, is also crucial. However, further multi-center prospective study with large sample size and controlling for all potential confounders is needed to better understand the mechanisms underlying these detrimental effects and develop targeted interventions.

## Appendices

**Table 4:** Comparison of baseline characteristics of the participants based on their diagnosis of NDR-AKI among non-critically ill hospitalized medical patients at St. Paul’s Hospital Millennium Medical College (SPHMMC), Ethiopia, from July 2019 to January 2022 (n=300) HIV: Human Immunodeficiency Virus, TB: Tuberculosis, DVT/PE: Deep Vein Thrombosis/Pulmonary Embolism, TB: Tuberculosis, ACEI: Angiotensin-Converting Enzyme Inhibitor, ARBs: Angiotensin Receptor Blockers, PPIs: Proton Pump Inhibitors, WBC: White Blood Cell, Hg: Hemoglobin, Na: Sodium, K: Potassium, Cl: Chloride, CNS: central nervous system. *=statistically significant at p-value ≤ 0.05.

Variable		NDR-AKI		P-value
		No (n=207) N (%)	Yes (n=93) N (%)	
Age Category	<40	85 (41.1)	33 (35.5)	0.457
	40-59	65 (31.4)	28 (30.1)	
	>=60	57 (27.5)	32 (34.4)	
Sex	Male	112 (54.1)	53 (57.0)	0.642
	Female	95 (45.9)	40 (43.0)	
Hypertension	No	132 (63.8)	42 (45.2)	0.003*
	Yes	75 (36.2)	51 (54.8)	
Diabetes	No	175 (84.5)	73 (78.5)	0.201
	Yes	32 (15.5)	20 (21.5)	
HIV	No	194 (93.7)	91 (97.8)	0.160
	Yes	13 (6.3)	2 (2.2)	
TB	No	185 (89.4)	89 (95.7)	0.072
	Yes	22 (10.6)	4 (4.3%)	
Cardiovascular disease	No	192 (92.8)	89 (95.7)	0.333
	Yes	15 (7.2)	4 (4.3)	
Chronic lung disease	No	185 (89.4)	88 (94.6)	0.142
	Yes	22 (10.6)	5 (5.4)	
Malignancy	No	184 (88.9)	90 (96.8)	0.025*
	Yes	23 (11.1)	3 (3.2)	
Sepsis	No	188 (90.8)	76 (81.7)	0.025*
	Yes	19 (9.2)	17 (18.3)	
Heart failure	No	158 (76.3)	68 (73.1)	0.551
	Yes	49 (23.7)	25 (26.9)	
Pneumonia	No	125 (60.4)	66 (71.0)	0.078
	Yes	82 (39.6)	27 (29.0)	
Hepatitis	No	194 (93.7)	83 (89.2)	0.178
	Yes	13 (6.3)	10 (10.8)	
CNS infection	No	189 (91.3)	93 (100.0)	0.003*
	Yes	18 (8.7)	0	
Stroke	No	181 (87.4)	88 (94.6)	0.059
	Yes	26 (12.6)	5 (5.4)	
DVT/PE	No	171 (82.6)	83 (89.2)	0.140
	Yes	36 (17.4)	10 (10.8)	
Cephalosporin	No	52 (25.1)	14 (15.1)	0.052
	Yes	155 (74.9)	79 (84.9)	
Penicillin	No	194 (93.7)	88 (94.6)	0.760
	Yes	13 (6.3)	5 (5.4)	
Macrolides	No	174 (84.1)	80 (86.0)	0.662
	Yes	33 (15.9)	13 (14.0)	
Quinolones	No	201 (97.1)	88 (94.6)	0.325
	Yes	6 (2.9)	5 (5.4)	
Vancomycin	No	132 (63.8)	50 (53.8)	0.101
	Yes	75 (36.2)	43 (46.2)	
Anti TB	No	185 (89.4)	88 (94.6)	0.142
	Yes	22 (10.6)	5 (5.4)	
Diuretics	No	154 (74.4)	34 (36.6)	<0.0001*
	Yes	53 (25.6)	59 (63.4)	
ACEI/ARBs	No	171 (82.6)	66 (71.0)	0.022*
	Yes	36 (17.4)	27 (29.0)	
Antiplatelet	No	191 (92.3)	90 (96.8)	0.139
	Yes	16 (7.7)	3 (3.2)	
PPIs	No	96 (46.4)	22 (23.7)	<0.0001*
	Yes	111 (53.6)	71 (76.3)	
Anticoagulants	No	91 (44.0)	42 (45.2)	0.847
	Yes	116 (56.0)	51 (54.8)	
Steroids	No	149 (72.0)	61 (65.6)	0.264
	Yes	58 (28.0)	32 (34.4)	
WBC	Normal	133 (64.3)	61 (65.6)	0.420
	Low	23 (11.1)	6 (6.5)	
	High	51 (24.6)	26 (28.0)	
Hg	Normal	98 (47.3)	31 (33.3)	0.026*
	Low	85 (41.1)	48 (51.6)	
	High	24 (11.6)	14 (15.1)	
Na	Normal	141 (68.1)	49 (52.7)	0.076
	Low	59 (28.5)	41 (44.1)	
	High	7 (3.4)	3 (3.2)	
K	Normal	167 (80.7)	59 (63.4)	<0.0001*
	Low	32 (15.5)	19 (20.4)	
	High	8 (3.9)	15 (16.1)	
Cl	Normal	137 (66.2)	45 (48.4)	0.014*
	Low	58 (28.0)	39 (41.9)	
	High	12 (5.8)	9 (9.7)	
